# Drug response is related to NR3C1 and FAAH polymorphism in Chinese pediatric epilepsy patients

**DOI:** 10.1186/s13052-025-01870-7

**Published:** 2025-02-07

**Authors:** Hongli Wang, Chu Li, Qian Li, Ning Li, Huiling Qin

**Affiliations:** 1 Department of Paediatric, Affiliated Hospital of Chengdu University, Chengdu, 610081 China; 2 The First Clinical Medical School, Guangzhou University of Chinese Medicine, Guangzhou, 510000 China; 3 Department of Paediatric, Dongying People’s Hospital, No. 317, Dongcheng South 1 Road, Dongying, 257091 China; 4 Department of Surgery, Guangrao County Traditional Chinese Medicine Hospital, Dongying, 257399 China; 5 Department of Rehabilitation Medicine, The Affiliated Hospital of Youjiang Medical University for Nationalities, No. 18, Zhongshan 2 Road, Youjiang District, Baise, 533000 China; 6 Key Laboratory of Research and Development On Clinical Molecular Diagnosis for High-Incidence Diseases of Baise, Baise, 533000 China

**Keywords:** NR3C1 rs41423247, FAAH rs324420, Drug response, Epilepsy, Liver dysfunction

## Abstract

**Background:**

Childhood epilepsy is a common neurological syndrome with complex etiology and recurrent seizures. It seriously affects the growth and development of child patients.

**Methods:**

NR3C1 rs41423247 and FAAH rs324420 polymorphisms were detected by the polymerase chain reaction in 105 pediatric epilepsy patients. Patients were divided into the good response group and the poor response group after anti-seizure medications (ASMs) treatment. According to the results of the liver function test (LFT), patients were divided into the no LFT disturbance group and the LFT disturbance group. Hardy–Weinberg balance was applied to analyze the population representation. The correlations were calculated by logistic regression analysis.

**Results:**

NR3C1 rs41423247 genotype and allele frequencies differed significantly between good response and poor response groups, while FAAH rs324420 did not. The CG genotype and C allele of NR3C1 rs41423247 were associated with good drug response, and the association was also detected in the dominant model. In addition, polymorphisms in NR3C1 and FAAH were not associated with liver damage induced by epilepsy medication.

**Conclusion:**

The polymorphism of NR3C1 rs41423247 might influence the drug response of epilepsy children.

## Introduction

Epilepsy is a disorder in which patients experience sudden, brief, and recurrent seizures of symptoms and/or signs, caused by a variety of etiological factors that result in abnormal or excessive firing activity of neurons in the brain [1]. Seizures, in the form of altered consciousness, involuntary motor, sensory, or psychiatric events, are a common chronic disease of the nervous system and are a significant cause of disability and death [2]. Since there is still a lack of effective preventive measures and cures for epileptic disorders at this stage, most patients require regular long-term drug therapy. Antiepileptic drugs are used to control the frequency of epileptic seizures and can be used as a single drug, but they are usually administered in combination with multiple drugs [3]. Most anti-seizure medications (ASMs) have different degrees of adverse effects, with dose-related adverse effects being the most common, including tremors, anorexia, nausea, vomiting, and drowsiness [4]. Long-term use is difficult for patients to accept, and therefore is prone to interruptions in treatment and recurrence of the disease. The ineffectiveness of antiepileptic treatment affects as many as 30% of epileptic patients, which is particularly important in the clinical treatment of epilepsy [5, 6]. The role of genetic diversity in the pathogenesis of epilepsy drug resistance seems indisputable [7]. Accordingly, this research spotlighted the roles of genetic polymorphism in treating epilepsy.

Single-nucleotide polymorphisms (SNPs) refer to single-nucleotide variations in a genetic sequence among people, which are the most frequent nucleotide variations in the human genome. SNPs are associated with epilepsy, which can influence the occurrence and therapy efficacy of epilepsy. The last decade has observed a significant improvement in unraveling several mutations in the genes that are responsible for drug resistance and associated adverse drug reactions in the treatment of epilepsy [8]. For example, the G allele of rs1491974 or rs6798347 of microglial P2Y12 receptors may elevate the risk of the frequency of seizure [9]. The rs57095329 SNP of miR-146a and rs3789243 of ATP-binding cassette subfamily B member 1 influence the drug response and resistance of epilepsy, which may guide the clinical treatment [10, 11]. These investigations unveil that SNPs play crucial roles in epilepsy. These findings would help the concerned neurologists to prescribe the medicine in epilepsy patients with more accuracy and to achieve the maximum therapeutic benefit. This would also help to improve the quality of life of patients with epilepsy and will avoid the recurrence of seizures.

Glucocorticoid receptor (NR3C1) is a gene encoded glucocorticoid receptor. The rs41423247 locus of NR3C1 carries a significantly higher frequency of the G allele in patients with functional seizures and major depression [12]. Mutations in fatty acid amide hydrolase (FAAH) (C385A; rs324420) have been associated with alterations in fronto-amygdala function, which may be associated with anxiety and fear symptoms [13]. COMT rs4680, FAAH rs324420, and OPRM1 rs1799971 models are related to the response of a placebo to nerve pain and can predict the effect of a placebo [14]. FAAH C384A genotype is associated with the risk of generalized epilepsy in Iranians [15]. Nowadays, the SNPs in NR3C1 and FAAH on the drug response of epilepsy remain unclear.

In this observation, the polymorphism of the rs41423247 locus on the NR3C1 gene and the rs324420 locus on the FAAH gene in children with epilepsy were detected, and the relationship between them and drug response in children with epilepsy was analyzed, so as to understand the clinical significance of these genetic polymorphisms in improving treatment outcomes for children with epilepsy.

## Materials and methods

### Participants

105 epileptic patients who were diagnosed and treated by Dongying People’s Hospital from June 2021 to October 2023 and met the criteria for admission and discharge were selected. This study has been approved by the Ethics Committee of Dongying People’s Hospital. All subjects signed informed consent before entering the group to collect blood samples. To protect patient privacy, only authorized medical personnel are allowed to access patient data. And the medical staff strictly follow the privacy policies established by the hospital.

Epilepsy is diagnosed by electroencephalography (EEG) examination results or typical seizure history. The diagnostic criteria refer to the Practical Clinical Definition of Epilepsy published by ILAE in 2014 The included criteria were: (1) receiving ASM treatment over 12 months; (2) aged from 2 years old to 17 years old, and (3) no consanguineous relationship between the subjects and no history of intermarriage. Patients with the following characteristics were excluded from the study: 1) comorbidity with other psychiatric disorders, 2) pseudoseizures, 3) unreliable seizure frequency without continuous electroencephalographic (CEEG) monitoring, 4) comorbidity with liver disease that can affect our detection indicators and failure of vital organs that can affect the life safety, 5) non-compliance with ASM treatment, and 6) incomplete clinical records.

Drug response to ASM therapy in patients with epilepsy was determined according to the International League Against Epilepsy (ILAE) definitions. A good drug response indicated completely seizure-free patients based on regular follow-up for at least 1 year during monotherapy or combination therapy at the best tolerated therapeutic dose [16, 17]. Patients with an adverse response are those who have been correctly medicated at the maximum tolerated dose for at least 12 months after monotherapy or combination therapy, with ineffectiveness and persistent seizures.

Liver function tests (LFT), including alanine aminotransferase, aspartate aminotransferase, alkaline phosphatase, total bilirubin, direct bilirubin, and so on, were performed to assess liver function at baseline and outpatient clinic follow-ups at 12 months after ASM treatments.

### DNA isolation from blood samples

DNA was extracted using a blood genome column rapid extraction kit (CWBIO, Taizhou, China) with an additional isopropanol precipitation step for optimal DNA quality. 200 μl of blood was added to a centrifuge tube, and 20 μl of Proteinase K and 200 μl Buffer GL was added successively. After incubating at 80℃ for 10 min, 200 μl isopropanol and 20 μl magnetic beads were added and mixed upside down. The centrifuge tube was fixed on a magnetic rack and left to stand for 1 min and the supernatant was aspirated and discarded. Then, buffer GW1 was added to the centrifuge tube for repeated washes of the magnetic beads. The wash solution was discarded. Finally, the eluent was added, mixed, and transferred to a new centrifuge tube. The desired DNA solution was collected and stored at −20°C.

The DNA sample quality was described by NanoDrop (Thermo Scientific, Willmington, USA), and the DNA sample with A260/A280 between 1.7 and 1.9 met the experimental requirements.

### Detection of target genotype

Specific site amplification primers were designed according to the target site sequence, and the polymorphisms of FAAH and NR3C1 genes were analyzed by the dideoxy terminal termination method (Sanger method). PCR amplification reaction system included: PCR master mix (Takara, Shiga, Japan) 25 μl, Forward Primer 1 μl, Reverse Primer 1 μl, template 1 μl, and ddH_2_O 22 μl. PCR reaction procedure was: pre-denaturation at 94℃ for 5 min; 28 circles of denaturation at 94℃ for 0.5 min, annealing at 58℃ for 0.5 min, extension at 72℃ for 1 min; and finally, 72°C extended for another 10 min. The primers of rs41423247 and rs324420 are listed in Table 1. The specificity of primers was verified by showing a single band of the correct amplified fragment size via agarose gel electrophoresis of the PCR products. All reagents, except for the template DNA, were prepared in an isolated pre-PCR room to prevent contamination. The PCR production was purified and put on the 3730 xl sequencer (Applied Biosystems, Foster City, USA) was used for sequencing. The Sequencing results will be analyzed by the DNA Chromas Analysis software (Technelysium, South Brisbane, Queensland).

**Table 1:** Primer sequences of FAAH and NR3C1

SNP ID	Gene Symbol	Nucleotide Change Location	Forward Primer	Reverse Primer
rs41423247	NR3C1	646 C > G	5′TGCTGCCTTATTTGTAAATTCGT 3′	5′ AAGCTTAACAATTTTGGCCATC 3′
rs324420	FAAH	385 C > A	5′ TGTTGCTGGTTACCCCTCTC3′	5′ CCCAAAATGACCCAAGATGC3′

*SNP* single nucleotide polymorphism

### Statistical analysis

SPSS 25.0 statistical software was applied to the study data for statistical processing (testing level *P* < 0.05 was considered statistically significant). Measurement data with normal distribution were presented as mean ± standard deviation (SD). The Categorical variables were expressed as number or percentage, and the χ2 test was used for comparison between groups, and the genetic specific risk was estimated as odds ratios (ORs) with associated 95% confidence intervals (CIs). Hardy–Weinberg balance was used to verify the population representation of target genes. And HWE deviations in control cohorts are frequently caused by a relatively small sample size, and expanding the sample size is desirable.

## Results

### General clinicopathological characteristics

A total of 105 patients were included in the analysis, including 50 males and 55 females, aged (9.54 ± 3.04) years (Table 2). There were 13 patients with epilepsy history, accounting for 12.4% (Table 2). There were 59 patients with good drug reactions, accounting for 56.2, and 46 patients with poor drug reactions, accounting for 43.8% (Table 2).

**Table 2:** Clinical Characteristics of Patients with Epilepsy

Items	Patients with Epilepsy (*N* = 105)
Age (years)	9.54 ± 3.04
Gender	
Male (N, %)	50, 47.6
Female (N, %)	55, 52.4
Family History of Epilepsy	
Yes (N, %)	13, 12.4
No (N, %)	92, 87.6
Drug Response	
Good (N, %)	59, 56.2
Poor (N, %)	46, 43.8

### Polymorphism detection results

Sequencing of target gene loci in 105 epilepsy patients showed that 8 cases of NR3C1 rs41423247 were wild type (CC) and 97 cases had gene mutations, including 32 cases of heterozygous mutations (CG) and pure 65 cases of combined mutation (GG). The NR3C1 646 C > G genotype frequency was tested to be consistent with Hard-Weinberg equilibrium (*P* = 0.10, Table 3).

**Table 3:** Distribution of rs41423247 and rs324420 in Patients with Epilepsy

SNP ID	N, %
rs41423247	
CC	8, 7.6
CG	32, 30.5
GG	65, 61.9
C	44, 21.0
G	166, 79.0
* P*^HWE^	0.10
rs324420	
AA	4, 3.8
AC	20, 19.1
CC	81, 77.1
A	28, 13.3
C	182, 86.7
* P*^HWE^	0.07

*HWE* Hardy–Weinberg equilibrium, *SNP* single nucleotide polymorphism

In all patients with epilepsy, 81 cases of FAAH rs324420 were wild type (CC), and 24 cases had gene mutations, including 20 cases of heterozygous mutations (CA) and 4 cases of homozygous mutations (AA). The rs324420 site complies with Hardy–Weinberg equilibrium (*P* = 0.07, Table 3).

### Correlation between target SNP and drug response

There was a statistically significant difference in the comparison of rs41423247 genotype frequencies between the good response and poor response groups (*P* < 0.001, Table 4). The frequency of the C allele was significantly higher in the good response group than in the poor response group (*P* < 0.001, Table 4). Significantly, χ^2^ analysis found that GG genotypes (OR = 6.023, 95% CI = 1.022–35.509, *P* = 0.047, Table 4) and C allele (OR = 4.822, 95% CI = 1.363–17.064, *P* = 0.015, Table 4) were associated with increased adverse drug reactions. The CG genotype increased the risk of poor response by over six times and the G allele elevated the risk of poor response by almost fivefold (Table 4).

**Table 4:** Distribution of rs41423247 and rs324420 in Patients with Epilepsy

SNP ID	Good response (*N* = 59)	Poor response (*N* = 46)	χ^2^	*P* value	OR (95%CI)	*P* value
rs41423247						
CC	6, 10.2	2, 4.3	/	/	1.00	
CG	27, 45.8	5, 10.9	/	/	0.721 (0.101–5.122)	0.744
GG	26, 44.1	39, 84.8	18.397	< 0.001	6.023 (1.022–35.509)	0.047
C	36, 30.5	8, 8.7	/	/	1.00	
G	82, 69.5	84, 91.3	7.425	0.008	4.822 (1.363–17.064)	0.015
rs324420						
AA	3, 5.1	1, 2.2	/	/	1.00	
AC	10, 16.9	10, 21.7	/	/	0.581 (0.046–7.263)	0.674
CC	46, 78.0	35, 76.1	0.898	0.638	1.655 (0.536–5.114)	0.381
A	12, 10.2	16, 17.4	/	/	1.00	
C	106, 89.8	76, 82.6	1.167	0.280	1.902 (0.503–7.189)	0.343

*CI* confidence interval, *OR* odds ratio, *SNP* single nucleotide polymorphism

No difference in rs324420 genotypes and alleles was found between the good response and poor response groups and no correlations were observed between this polymorphism and drug response (All *P* > 0.05, Table 4).

### Inheritance genotype models of different drug responses

The genotype model of inheritance of rs41423247 and rs324420 was exhibited in Table 5. In genetic model analysis, the dominant model of rs41423247 showed a difference between the good response group and the poor response group (*P* < 0.001, Table 5) and it was related to the good drug response (OR = 8.253, 95% CI = 2.784–24.467, *P* < 0.001, Table 5). The recessive model of rs41423247 together with the dominant and recessive models of rs324420 represented no correlation with the drug response of ASMs (All *P* > 0.05, Table 5).

**Table 5:** Distribution of genetic model of rs41423247 and rs324420 in Patients with Epilepsy

Model	Genotype	Good response (*N* = 59)	Poor response (*N* = 46)	χ^2^	*P* value	OR (95%CI)	*P* value
	rs41423247						
Dominant	CC-CG	33, 55.9	7, 15.2	/	/	1.00	
	GG	26, 44.1	39, 84.8	18.169	< 0.001	8.253 (2.784–24.467)	< 0.001
Recessive	CC	6, 10.2	2, 4.3	/	/	1.00	
	CG-GG	53, 89.8	44, 95.7	1.245	0.461	1.826 (0.280–11.921)	0.529
	rs324420						
Dominant	AA-AC	13, 22.0	11, 23.9	/	/	1.00	
	CC	46, 78.0	35, 76.1	0.052	0.820	1.527 (0.507–4.605)	0.452
Recessive	AA	3, 5.1	1, 2.2	/	/	1.00	
	AC-CC	56, 94.9	45, 97.8	0.598	0.630	0.333 (0.023–4.717)	0.416

*OR* odds ratio, *SNP* single nucleotide polymorphism

### Relationship between polymorphisms and the risk of liver dysfunction

Of the genotyped patients, 39 cases experienced hepatotoxicity while 66 did not. Individuals with the variant of the rs41423247 and rs324420 represented no statistical significance of LFT impairment (All *P* > 0.05, Table 6).

**Table 6:** Distribution of rs41423247 and rs324420 in patients with epilepsy

SNP ID	No LFT disturbance (*N* = 66)	LFT disturbance (*N* = 39)	χ^2^	*P* value	OR (95%CI)	*P* value
rs41423247						
CC	3, 4.5	5, 12.8	/	/	1.00	
CG	19, 28.8	13, 33.3	/	/	3.950 (0.832–18.750)	0.084
GG	44, 66.7	21, 53.8	3.020	0.221	1.418 (0.572–33.519)	0.451
rs324420						
AA	3, 4.5	1, 2.6	/	/	1.00	
AC	9, 13.6	12, 30.8	/	/	0.886 (0.085–9.223)	0.920
CC	54, 81.8	26, 66.7	4.589	0.101	2.990 (1.099–8.131)	0.032

*CI* confidence interval, *LFT* liver function test, *OR* odds ratio, *SNP* single nucleotide polymorphism

## Discussion

This study investigated the relationship between drug response to epilepsy treatment and the rs41423247 polymorphism on the NR3C1 gene or the SNP rs324420 on the FAAH gene. The genotypes and allele frequencies of the rs41423247 locus on the NR3C1 gene and the rs324420 locus on the FAAH gene were found to be in accordance with the Hardy–Weinberg balance in 105 epileptic patients. Of the 105 patients who completed ASM treatment in this study, 59 had a good response to the ASMs, and 46 responded negatively. In the good response group, the CG genotype frequency, the C allele frequency, dominant genotype at the rs41423247 locus on the NR3C1 gene were significantly higher than in the poor response group. However, there was no significant difference in rs324420 polymorphism of the FAAH gene between the two groups. Rs41423247 polymorphism on the NR3C1 gene might be associated with drug response to ASM treatment, and antiepileptic efficacy was better in patients with the C allele and CG genotype. In addition, the rs41423247 polymorphism on the NR3C1 gene and the rs324420 on the FAAH gene were not associated with liver injury after epilepsy drug therapy. The findings indicated that the liver function of epilepsy cases after drug therapy might not be influenced by different genotypes of rs41423247 and rs324420 polymorphisms. However, other factors, such as medication type and duration of treatment, may be confounding factors that were not considered in the present study, which should be verified in future studies.

Epilepsy is a chronic disease caused by abnormal discharges of neurons in the brain. Patients may experience sudden loss of consciousness, foaming at the mouth, convulsions, muscle stiffness, or tremors during an attack [18]. The causes of epilepsy are varied, including genetic factors, brain infections, traumatic brain injuries, brain tumors, and so on [19]. Non-pharmacological treatments such as epilepsy surgery, neuromodulation, and ketogenic diets have made great strides, but pharmacological treatments are still the mainstay [20]. Antiepileptic drugs can control seizures by modulating neuronal excitability and inhibiting neuronal over-discharge [21]. Phenobarbital is the oldest antiepileptic drug still in wide clinical use. These drugs successfully suppress seizures in the majority of patients. However, in approximately 20–40% of patients, epilepsy is drug resistant [22].

The therapeutic response to ASMs varies significantly in different individuals, and blood concentrations at conventional doses can exceed the range of effective therapeutic concentrations, leading to therapeutic failure, reduced tolerance, and adverse effects. Genetic polymorphisms are one of the main reasons for these differences. Therefore, this article focused on the polymorphism in the drug response of treatment in epilepsy in order to explore the mechanism of drug response in ASM, so as to assist personalized treatment strategies. Many genetic polymorphisms play a role in the pathogenesis and treatment of epilepsy, e.g., SCN1A ABCG2, SCN1A, CYP3A5, and SCN2A [23–25]. The rs211037 polymorphism on the GABRG2 gene is associated with valproic acid-induced adverse drug reactions, and the CC genotype was associated with the absence of seizures after treatment [26]. The rs2556375 of BCL11A increases the seizure susceptibility and risk of drug resistance [27]. The SNP of NR3C1 is correlated with difficult-to-treat rhinosinusitis, glucose metabolism type 2 diabetes, and IgA nephropathies [28–30]. Notably, NR3C1 rs41423247 polymorphism is related to functional seizures of Iranian [12]. In our study, we found a correlation between NR3C1 and drug response to ASM treatment in Chinese pediatric epilepsy patients, mainly in the form of CG genotype, and C allele is associated with a good response to the drug, suggesting that the NR3C1 rs41423247 polymorphism may affect the therapeutic effect of ASM in epilepsy patients. The role of glucocorticoid receptors in the nervous system may influence epileptic drug response. Glucocorticoids can regulate the release of neurotransmitters, neuronal excitability and synaptic plasticity. The functional status of the glucocorticoid receptor encoded by the NR3C1 gene may affect the regulatory effect of glucocorticoids on the nervous system, and thus affect the efficacy of epilepsy drugs. In addition, the polymorphism of NR3C1 gene may affect the metabolism and transport of epilepsy drugs. Different NR3C1 gene polymorphisms may lead to changes in the structure and function of glucocorticoid receptors, thereby affecting drug metabolism and transport. Some studies have suggested that polymorphisms in the NR3C1 gene may indirectly affect the metabolism and transport of epilepsy drugs by affecting the function of the hypothalamic–pituitary–adrenal axis (HPA) [31]. However, the mechanism should be verified in future studies. In the clinical treatment of patients with epilepsy, understanding the genotype of the NR3C1 rs41423247 site can help doctors develop a more personalized treatment plan. For carriers of the CG genotype, doctors may be more inclined to choose conventional antiepileptic drugs during initial treatment. Because these patients may have better anti-epileptic efficacy according to the conclusion of the study, they can avoid overuse of some powerful drugs that may have more side effects. For carriers of the CC gene, doctors can consider adjusting treatment options, such as changing drug types, adjusting drug dosages, or combining other drugs. In addition, accumulating data supports an autoimmune basis in patients with antiepileptic drug-resistant seizures [32, 33]. NR3C1 has been reported to serve as potential immune-related biomarkers [34]. And NR3C1 is linked with a deregulated hypothalamus–pituitary–adrenal (HPA) axis and psychopathology [35]. Therefore, the role of NR3C1-mediated immune response in epilepsy drug response is worth exploring in depth. Besides, there is a close correlation between probiotic-mediated immunity and drug resistance in epileptic patients [36]. Probiotics may have a positive impact on drug resistance in patients with epilepsy by regulating the immune system, enhancing the intestinal barrier, regulating intestinal microbiota, and other mechanisms [37]. In the clinical treatment of epilepsy patients, probiotics supplement therapy has important research significance.

FAAH rs324420 is widely researched in several diseases or physiological processes, such as motor performance, memory fading, and susceptibility to methamphetamine dependence [38–40]. In the Iranian population, FAAH rs324420 genotype and allele distribution were shown to be associated with generalized epilepsy and not with focal epilepsy [15]. In our study, the FAAH rs324420 polymorphism did not correlate with drug response after ASM treatment in patients with epilepsy These results suggest that polymorphisms in FAAH do not correlate with drug response to ASM therapy for epilepsy. However, the sample size of this study is relatively small, and the comparison of polycentric large samples of homogenous populations is lacking. Although relatively few direct studies have been conducted on the role of FAAH in patients with different types of epilepsy, its role in epilepsy warrants further investigation because it is a key enzyme in the endocannabinoid system [41]. And the endocannabinoid system plays an important role in neuropsychiatric diseases [42]. The study showed that FAAH polymorphism is not associated with epilepsy resistance, which may mean that FAAH gene polymorphism is not involved in these key drug action links in the mechanism of epilepsy resistance. On the other hand, it is also possible that our study sample is small and there is a certain result bias. Therefore, in the future, large-scale clinical studies can be conducted to collect NR3C1 and FAAH gene information and epilepsy drug treatment response data, and analyze the relationship between NR3C1 and FAAH gene polymorphisms and epilepsy drug response. In addition, the relationship of NR3C1 and FAAH with epileptic drug response can be comprehensively evaluated in combination with other biological indicators or genome interactions. Then explore the personalized epilepsy treatment strategy based on NR3C1 and FAAH. In addition, other external factors such as diet and environmental exposures may influence genetic expression and drug metabolism, potentially interacting with the polymorphisms studied. But they were not included in the current study. Thus, future studies should take into account external factors and observe the relationship between NR3C1 and FAAH genetic polymorphisms and epilepsy drug response after controlling for these factors.

Interestingly, the present results also demonstrated that both NR3C1 and FAAH polymorphisms showed no significant correlation with liver injury associated with ASM treatment. Antiepileptic drugs are mainly metabolized by cytochrome P450 enzyme series (CYP) or glucosylation reaction. The polymorphism of NR3C1 and FAAH genes may not affect the metabolic process of antiepileptic drugs in the liver, and thus have nothing to do with liver injury. Some current studies have found that probiotics can improve liver function in patients with epilepsy [43]. This may be because probiotics improve gut microbiota outcomes and reduce the production of inflammatory mediators, thereby reducing the burden of inflammation on the liver. Signaling molecules produced by probiotics may affect NR3C1 gene expression [44]. This regulatory effect of probiotics may vary in individuals with different polymorphisms of the NR3C1 gene. FAAH gene polymorphism may also affect the metabolism of probiotics. Different FAAH genotypes may lead to differences in the endocannabinoid system, which in turn affect the physiological function of the gut. In addition, NR3C1 gene polymorphism may affect the body's ability to regulate inflammation, and thus affect liver function [45]. Therefore, inflammatory responses may mask the potential effects of genetic polymorphisms on liver function. Therefore, exploring the role of systemic inflammation in the relationship between gene polymorphism and liver function is helpful to understand the influencing factors of liver function more comprehensively.

## Conclusion

In consequence, NR3C1 rs41423247 polymorphism may be related to the response to anti-epileptic drugs, and G allele and CG genotype carriers have better anti-epileptic efficacy. FAAH rs324420 polymorphism was not associated with drug response to epilepsy treatment. At the same time, NR3C1 rs41423247 and FAAH rs324420 polymorphisms were not associated with liver injury in epilepsy treatment. These findings provide new ideas and methods for personalized treatment of epilepsy.

## Data Availability

Corresponding authors may provide data and materials.
